# Predicting individual plant performance in grasslands

**DOI:** 10.1002/ece3.3393

**Published:** 2017-09-22

**Authors:** Katharina Herz, Sophie Dietz, Sylvia Haider, Ute Jandt, Dierk Scheel, Helge Bruelheide

**Affiliations:** ^1^ Institute of Biology/Geobotany and Botanical Garden Martin Luther University Halle‐Wittenberg Halle Germany; ^2^ Leibniz Institute of Plant Biochemistry Halle Germany; ^3^ German Centre for Integrative Biodiversity Research (iDiv) Halle‐Jena‐Leipzig Leipzig Germany

**Keywords:** biodiversity exploratories, community‐weighted means, functional diversity, local neighborhood, phytometer, plant performance

## Abstract

Plant functional traits are widely used to predict community productivity. However, they are rarely used to predict individual plant performance in grasslands. To assess the relative importance of traits compared to environment, we planted seedlings of 20 common grassland species as phytometers into existing grassland communities varying in land‐use intensity. After 1 year, we dug out the plants and assessed root, leaf, and aboveground biomass, to measure plant performance. Furthermore, we determined the functional traits of the phytometers and of all plants growing in their local neighborhood. Neighborhood impacts were analyzed by calculating community‐weighted means (CWM) and functional diversity (FD) of every measured trait. We used model selection to identify the most important predictors of individual plant performance, which included phytometer traits, environmental conditions (climate, soil conditions, and land‐use intensity), as well as CWM and FD of the local neighborhood. Using variance partitioning, we found that most variation in individual plant performance was explained by the traits of the individual phytometer plant, ranging between 19.30% and 44.73% for leaf and aboveground dry mass, respectively. Similarly, in a linear mixed effects model across all species, performance was best predicted by phytometer traits. Among all environmental variables, only including land‐use intensity improved model quality. The models were also improved by functional characteristics of the local neighborhood, such as CWM of leaf dry matter content, root calcium concentration, and root mass per volume as well as FD of leaf potassium and root magnesium concentration and shoot dry matter content. However, their relative effect sizes were much lower than those of the phytometer traits. Our study clearly showed that under realistic field conditions, the performance of an individual plant can be predicted satisfyingly by its functional traits, presumably because traits also capture most of environmental and neighborhood conditions.

## 1 INTRODUCTION

Plant functional traits are widely used to describe ecological functions and strategies of plants (Freschet, Cornelissen Johannes, van Logtestijn Richard, & Aerts, 2010; Violle et al., 2007). Fast‐growing species are for example characterized by a high specific leaf area (SLA), and high leaf nitrogen and phosphorus contents (Freschet et al., 2010; Pérez‐Harguindeguy et al., 2013). In a meta‐analysis, de Bello et al. (2010) identified trait‐service clusters, which are groups of functional traits and their correlating multiple ecosystem services. Although most studies use functional traits to predict ecological functions at the community level, some efforts have also been made to understand the relationships between functional traits and individual plant performance. For example, Gross et al. (2009) found that growth responses of individual plants are related to the SLA of the community in subalpine grasslands. Moreover, trait combinations that maximize plant growth were well predictable by individual‐centered models in a study of Maire et al. (2013). Still, in purpose of understanding relationships between measurable plant characteristics, quantifying the relationships between plant functional traits and individual plant performance is a current issue in ecology (Ackerly, Dudley, Sultan, Schmitt, & Coleman, 2000; Geber & Griffen, 2003; Violle et al., 2007). As long‐term monitoring of plant sizes and biomasses requires considerable efforts, easily measurable functional traits would provide highly desirable proxies for individual plant performance.

Plant performance is not only associated with functional traits but also linked to abiotic conditions (Aerts & Chapin, 2000). In grasslands, these comprise climate and soil properties and land‐use intensity. Although land‐use intensity cannot easily be quantified, a practical approach has been developed in the German Biodiversity Exploratories. Thereby, the frequency of mowing, grazing, and fertilization was integrated into a land‐use intensity Index (LUI; Blüthgen et al., 2012). LUI has been found to be a potent predictor for nutrient concentrations of aboveground biomass (Blüthgen et al., 2012; Klaus et al., 2011), relative growth rates (Breitschwerdt et al. unpublished), and ecosystem functions (Allan et al., 2015). However, whether land‐use intensity has positive or negative effects on individual plant performance strongly depends on the competitive ability and disturbance tolerance of the focal species.

In addition to characteristics of the focal plant and the abiotic environment, the surrounding community may also affect plant performance. To test such neighborhood impacts, grasslands are very suitable study systems as they show a high species richness at small spatial scale (Wilson, Peet, Dengler, Pärtel, & Palmer, 2012). Neighborhood conditions can be described using functional traits of all plant individuals growing in the neighborhood of a focal plant, exerting an impact as either mean or variation of trait values. Community‐weighted mean traits (CWM) weigh the traits of all neighbors by their abundance (Garnier et al., 2004), and thus reflect the most abundant trait values. In contrast, functional diversity (FD) describes trait dissimilarity among the neighborhood species. Comparing CWM, FD, and several other diversity measures, Fu et al. (2014) found that CWM had a two times higher explanatory power than FD for community productivity. However, such neighborhood effects on single individuals were so far often investigated in forests (e.g., Canham et al., 2006; Kröber et al., 2015; Uriarte, Canham, Thompson, & Zimmerman, 2004) but rarely in grassland species (e.g., Kraft, Godoy, & Levine, 2015; Le Bagousse‐Pinguet et al., 2015).

Using a phytometer approach (Clements & Goldsmith, 1924; Dietrich, Nilsson, & Jansson, 2013), we aimed at finding the most important predictors for individual plant performance in grasslands. We expected that functional plant traits also capture environmental and neighborhood conditions as a plant individual's traits reflect the conditions it was subjected to during its life cycle. Thus, we hypothesized that performance can be predicted best by the traits of the focal phytometer plant, followed by other factors including environmental variables, CWM, and FD of the local neighborhood, accounting for additional effects (e.g., exudates, microbial rhizosphere interactions) on performance not captured by traits.

## 2 MATERIAL AND METHODS

In 2014, we set up an experiment in the grassland plots of the three German Biodiversity Exploratories (Schorfheide‐Chorin, Hainich, and Schwäbische Alb) (Fischer et al., 2010). Of 50 plots available per Exploratory, 18 were selected differing in land use (six meadows, pastures, and mown pastures) and land‐use intensity. The land‐use intensity of the Biodiversity Exploratories was summarized by an index calculated according to formula (1)

(1) $${\text{LUI}}_{i}=\sqrt{\frac{F_{i}}{F_{R}}+\frac{M_{i}}{M_{R}}+\frac{G_{i}}{G_{R}}}$$

where *F*_*i*_ is the fertilization level in kg nitrogen per ha and year, *M*_*i*_ the mowing frequency per year, and *G*_*i*_ the grazing intensity defined as livestock units days of grazing per ha and year on each plot *i*, related to the mean values of *F*_*R*_, *M*_*R*_ and *G*_*R*_ of each of the three Exploratory regions *R* (Blüthgen et al., 2012). We used mean LUI values between 2006 and 2014 for our analyses. In addition to LUI, each plot was described by further environmental variables such as air temperature at 10 cm and 200 cm aboveground, relative humidity at 200 cm aboveground, and soil moisture at 10 cm depth. We calculated the mean of each of these variables for our study period from May 2014 to July 2015 from monthly mean values. Soil characteristics were described by NaHCO_3_‐extractable P (plant‐available P), pH, and total P, C, and N. Both climate and soil variables were available for every plot (see Klaus et al. (2016) and Schöning et al. (2013) for soil sampling methods and analyses of total C and N; Hedley, Stewart & Chauhan (1982) and Alt, Oelmann, Herold, Schrumpf & Wilcke (2011) for analyses of pH and plant‐available P; and Raessler, Rothe & Hilke (2005) for analyses of total P). Climate was recorded in weather stations in the center of each Exploratory plot, and soil data were acquired through a central soil sampling campaign in 2014.

Ten grass and ten forb species were planted into every plot as phytometers: *Alopecurus pratensis* L., *Anthoxanthum odoratum* L., *Arrhenatherum elatius* (L.) P.Beauv. ex J.Presl & C.Presl., *Cynosurus cristatus* L., *Dactylis glomerata* L., *Festuca pratensis* Huds., *Helictotrichon pubescens* (Huds.) Schult. & Schult.f., *Lolium perenne* L., *Poa pratensis* L., *Poa trivialis* L. (all Poaceae), *Achillea millefolium* L., *Bellis perennis* L., *Centaurea jacea* L. (Asteraceae), *Galium mollugo* L., *Galium verum* L. (Rubiaceae), *Plantago lanceolata* L. (Plantaginaceae), *Ranunculus acris* L., *Ranunculus bulbosus* L. (Ranunculaceae), *Rumex acetosa* L. (Polygoncaceae), *Veronica chamaedrys* L. (Plantaginaceae). These selected perennial species were among the most frequent and abundant species in all grassland plots of the Exploratories and thus can be considered characteristic for these grasslands. Raising of the phytometers was performed in the greenhouse of the Botanical Garden Halle (Germany) from December 2013 till April 2014. Seeds from 11 species were collected from the grasslands in the Exploratories' regions while seeds from nine species were ordered from commercial suppliers (see also Herz et al., 2017). Planting of phytometers took place from May to June 2014. Every species was planted into each of the 18 selected plots per Exploratory, resulting in a total of 54 individuals per species.

In May 2015, the neighborhood vegetation was recorded in a circle of 15 cm radius (Bittebiere & Mony, 2014) around each phytometer by estimating the percentage cover of every plant species occurring within the circle. Abundances below 10% were estimated in 1% steps and above 10% in 5% steps, that is, 1%, 2%, 3%, 4%, 5%, 6%, 7%, 8%, 9%, 10%, 15%, 20%, and 25% and so on. From June to July 2015, we harvested one individual of every planted phytometer species per plot and determined performance by assessing the dry weight of roots, shoots, and leaves, resulting in a total of three performance measures (root, leaf, and aboveground dry mass) for every individual plant. Additionally, we sampled three individuals of all occurring neighborhood species in randomly selected plots. We were able to collect traits (Table 1) from species making up on average at least 80% of the total coverage of the local neighborhood, which is considered sufficient for CWM and FD analyses as pointed out by Garnier et al. (2004) and Li et al. (2017). CWM and FD were based on the neighbor species mean traits across all plots in which the neighbor plants were sampled. If possible, we used trait values that were specific for an Exploratory, to calculate CWM and FD values for the species occurring in the respective Exploratory. However, if there was a species occurring, for example, in all three Exploratories but only samples in two of them were gathered, we took the mean values of these two sites to calculate CWM and FD for the third not sampled Exploratory. The leaves and roots of all phytometers and neighbor plants were scanned with a HP Scanjet Flatbed Scanner at 600 dpi and analyzed with the programs WinFOLIA (Version 2004a) and WinRHIZO (Version 2008a). All parts were then dried at 60°C for 3 days and weighed again. Roots and leaves were ground to chemically analyze the C and N concentrations (vario EL cube from Elementar, Hanau, Germany), P concentration (photometric phosphate essay), and K, Mg, and Ca concentrations (atom absorption spectrometry with AAS vario 6 from Analytik Jena, Germany). For P, K, Mg, and Ca concentrations, it was necessary to conduct a digestion with nitric acid. Leaves of phytometer plants could not be analyzed chemically. A description of all functional traits is given in Table 1. For a more detailed description of the phytometer raising process, experimental setup and harvest methods see Herz et al. (2017).

**Table 1:** Summary and description of all traits and variables that were used as predictors for phytometer performance

Abbreviation	Unit	Description	Predictor type
LAR	cm²/g	Leaf area per unit total dry mass	PT, CWM, FD
LCaC	μmol/g	Leaf calcium concentration	CWM, FD
LCC	%	Leaf carbon concentration	CWM, FD
LCNR	g/g	Leaf carbon‐to‐nitrogen ratio	CWM, FD
LDMC	mg/g	Leaf dry mass per leaf fresh mass	PT, CWM, FD
LKC	μmol/g	Leaf potassium concentration	CWM, FD
LMgC	μmol/g	Leaf magnesium concentration	CWM, FD
LNC	%	Leaf nitrogen concentration	CWM, FD
LPC	μmol/g	Leaf phosphorus concentration	CWM, FD
RCaC	μmol/g	Root calcium concentration	PT, CWM, FD
RCC	%	Root carbon concentration	PT, CWM, FD
RCNR	g/g	Root carbon‐to‐nitrogen ratio	PT, CWM, FD
RDMC	mg/g	Root dry mass per root fresh mass	PT, CWM, FD
RKC	μmol/g	Root potassium concentration	PT, CWM, FD
RMgC	μmol/g	Root magnesium concentration	PT, CWM, FD
RMV	g/cm³	Root mass per unit root volume	PT, CWM, FD
RNC	%	Root nitrogen concentration	PT, CWM, FD
RPC	μmol/g	Root phosphorus concentration	PT, CWM, FD
RSR	g/g	Dry mass of roots per unit dry mass of aboveground organs	PT, CWM, FD
RVol	cm³	Root volume	PT, CWM, FD
SDMC	mg/g	Shoot dry mass per shoot fresh mass	
SLA	m²/kg	Leaf area per unit leaf dry mass	PT, CWM, FD
LUI		Land‐use intensity Index	Env
PAP	mg/kg	Total plant‐available phosphorus concentration	Env
pH		pH of soil	Env
Total C	g/kg	Total soil carbon concentration	Env
Total N	g/kg	Total soil nitrogen concentration	Env
Total P	g/kg	Total soil phosphorus concentration	Env
rH 200	%	Relative humidity 200 cm aboveground	Env
SM 10	% VWC	Soil moisture at 10 cm depth	Env
Ta 10	°C	Air temperature 10 cm aboveground	Env
Ta 200	°C	Air temperature 200 cm aboveground	Env

The last column shows for which of the four predictor types the trait was used. Total number of used predictors *n *= 78. CWM, community‐weighted mean; Env, environment; FD, functional diversity; PT, phytometer traits. RVol was not included to predict root biomass.

All statistical analyses were conducted in R (version 3.2.3, R Core Team, 2015; Vienna, Austria). Using the relative cover of each neighboring plant species around each phytometer, we calculated CWM and FD for each functional trait and performance measure. CWM values were obtained using the function matrix.t (package SYNCSA, Debastiani & Pillar, 2012) according to formula (2)

(2) $$\text{CWM}=\sum_{i=1}^{S}p_{i}t_{i}$$

where *S* is the number of species in a radius of 15 cm around each phytometer, *p*_*i*_ the relative cover, and *t*_*i*_ the trait values of a species *i*.

Functional diversity values were calculated using Rao's Q (Rao, 1982), using the function divc (package ade4, Dray & Dufor, 2007) according to formula (3):

(3) $$\text{FD}=\sum_{i=1}^{S-1}\sum_{j=\;i+1}^{S}p_{i}p_{j}d_{ij}$$

where *S* is the number of species in a radius of 15 cm around each phytometer, *p*_*i*_ and *p*_*j*_ the abundances of species *i* and *j*, respectively, and *d* the trait distance between species *i* and *j*. We took the square root of the trait distance as divc internally takes the square of distance values, see Champely & Chessel (2002).

We used variance partitioning to identify which predictor type (see Table 1) explained the highest amount of variation in the three performance variables of the phytometers (function varpart, package vegan, Oksanen et al., 2016). Therefore, we constructed a model for every species for each of the three response variables (performance) using (i) all phytometer traits (14 variables), (ii) all environmental variables (LUI, climate and soil conditions; 10 variables), (iii) all CWM (27 variables), and (iv) all FD (27 variables) of neighbor trait values as predictors (in total 78 variables), hereafter referred to as the four predictor types.

Prior to the analyses, we had to exclude values of root carbon content (RCC) below one (three samples), as they were caused by a wrong estimation of peak area by the C/N‐analyzer. We transformed RVol, RCaC, RMgC, RPC, RKC, RSR, LAR, RDMC, RMV, SLA, RNC, and RCC by natural logarithm and LDMC and RCNR by square root while performance variables were transformed by common logarithm to achieve normal distribution of the residuals (see Table 1 for abbreviations). Afterward, we excluded extreme outliers of all phytometer traits that exceeded three times the upper quartile. All predictors were scaled by mean and standard deviation. To check for correlations among predictors, we used a Pearson correlation matrix using the package corrplot (Wei & Simko, 2006; Figure S1). In particular, dry mass variables of CWM and their corresponding FD variables as well as root nitrogen content and root carbon‐to‐nitrogen ratio were highly correlated (Figure S1). Therefore, we chose the following statistical approach.

To find the most parsimonious combination of predictors for the performance of each phytometer species, we applied two model selection steps. At first, we used lasso selection of a generalized linear model using the glmnet package (Friedman, Hastie, & Tibshirani, 2010). This procedure is particularly useful in situations with numerous and potentially correlated predictor variables. We varied the effective degrees of freedom of the lasso (i.e., λ), using the cv.glmnet function and 100‐fold cross‐validation, thus identifying the λ and the corresponding predictor variables at which the mean cross‐validation error was minimal. In a second step, we used these identified variables as fixed factors in a linear mixed effects model (function lmer, package lmerTest, Kuznetsova, Brockhoff, & Haubo, 2015), including species and plot as crossed random factors (see Results in Table S1). The step function was employed to remove insignificant predictors (package lmerTest; Satterthwaite approximation). To evaluate the goodness of fit of the models, marginal and conditional *R*² values were calculated according to equations 26, 29, and 30 in Nakagawa and Schielzeth (2013). As model comparisons could only be made with a full data matrix of all predictor variables without missing values, we tested in a final step whether the model was still valid if we included additional data lines, which had missing values in one or more predictors but were complete with respect to the finally identified predictor variables (see Table S2).

## 3 RESULTS

**Figure 1:**
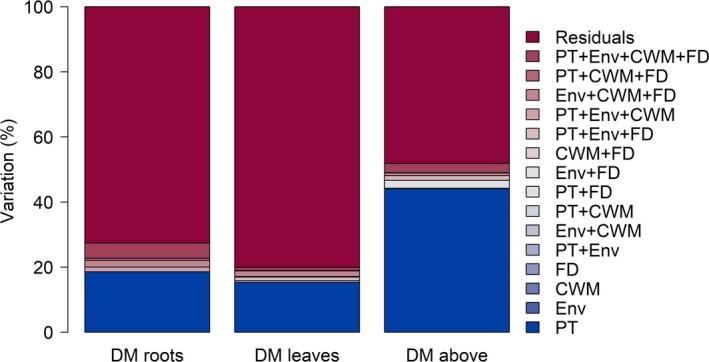
Variance partitioning. Stacked bars show how much variation (in %) in dry mass of roots, leaves, and aboveground organs was explained by which predictor type. DM, dry mass; PT, phytometer traits; Env, Environment; CWM, community‐weighted mean; FD, functional diversity. For exact values see Table S3

Among all four predictor types (Table 1), the traits of the phytometers explained most variation in performance variables with at minimum 19.3% (leaf dry mass) and at maximum 44.73% (aboveground dry mass; Figure 1, Table S3). Environmental variables and FD explained at maximum 1.13% and 0.43%, respectively, while CWM did not explain any variance (0%). The amount of unexplained variation ranged between 48.13% for aboveground dry mass and 80.06% for leaf dry mass (Figure 1). Furthermore, the amount of jointly explained variation did not exceed 4.71% for any performance variable.

The coefficient of determination of our models including both fixed and random factors (conditional *R*²) was 0.370 for root dry mass (Table 2), 0.572 for leaf dry mass, and 0.727 for aboveground dry mass. Marginal *R*² values (accounting only for fixed factors) were slightly lower and ranged between 0.309 for root dry mass and 0.700 for aboveground biomass.

**Table 2:** Results of the linear mixed effects models

Predictor	DM roots		DM leaves		DM above	
	Estimate	*p*	Estimate	*p*	Estimate	*p*
Intercept	−0.553	***	−1.144	***	−0.638	***
LAR	−0.056	**			−0.062	***
RCaC	0.049	**	0.063	*	0.050	**
RCC	−0.131	***			−0.134	***
RSR			−0.308	***	−0.467	***
SLA	−0.034	*	−0.200	***		
LUI					0.037	*
CWM_LDMC	−0.047	**			−0.047	**
CWM_RCaC	0.044	*			0.059	**
CWM_RMV	−0.037	*				
CWM_RVol			0.064	*		
FD_LKC			0.056	*		
FD_RMgC	−0.040	*				
FD_SDMC			−0.096	***		
*n* samples	346		346		346	
*R*² marg.	.308		.443		.700	
*R*² cond.	.363		.572		.727	

From the predictors shown in Table 1, we first selected the most parsimonious model by lasso procedure using 100‐fold cross‐validation (see Table S1) and then included them into a linear mixed effects model, using species and plot as random factors. From this model, we removed the insignificant predictors. All variables were scaled by mean and standard deviation prior to analyses. For abbreviations of predictors, see Table 1. RVol was not included to predict root biomass.

DM, dry mass.

*p < 0.05; **p < 0.01; ***p < 0.001

Between six and eight predictors remained in the final models of the three performance variables after the two selection steps (Table 2, Figure S2–S4). Root calcium concentration was the only predictor that occurred in the best models of all three performance variables and had a positive effect on growth in all cases. Root carbon concentration had the highest effects on root and leaf biomass, while aboveground biomass was best explained by root to shoot ratio. LUI, two CWM traits (RMV and RVol), and three FD traits (leaf potassium concentration [LKC], RMgC and shoot dry mass per shoot fresh mass [SDMC]) only occurred once in any of the three models. Apart from LUI, no other environmental variable emerged for any performance variable.

When using a higher number of samples by excluding only missing values and outliers of the variables selected by the lasso selection (Table S2), all predictors remained significant and in most cases, the relative effect sizes increased. There was also an increase in the conditional *R*² value of the models, except for aboveground dry mass.

## 4 DISCUSSION

To our knowledge, this is the first study investigating the effects on individual plant performance in grasslands including environmental variables and traits of the local neighborhood. From the 78 predictors, we were able to identify the six to eight most important ones for root, leaf, and aboveground biomass.

Across all variables and in accordance with our first hypothesis, plant performance was best predicted by the functional traits of the phytometers, compared to impacts of the environment and functional composition of the local neighborhood. This was shown both by variance partitioning and the low relative effect sizes of local neighborhood traits and their scarce representation in the linear mixed effects models. Thus, functional traits of phytometer plants were more important drivers of plant performance than environmental factors. However, another explanation could be that plant functional traits captured the environmental conditions the plants were subjected to better than our measured variables. The results on variance partitioning point to the first explanation, as there was no variance shared between phytometer traits and environmental variables.

Root carbon concentration had a strong negative relationship with root dry mass, which indicates that heavier roots contain higher oxidized carbon compounds, such as carbohydrates like starch or glucose rather than of more reduced and polymerized structural carbohydrates like lignin, cellulose or pectin (Poorter & Villar, 1997). In addition, heavier roots may contain to a higher degree other vital elements such as N, S, and P. Given that all phytometers were raised from seeds, root dry mass can also be taken as a measure of root growth. Root growth is lower when more reduced and polymerized carbon compounds are produced, which is explained by their higher construction costs compared to nonstructural carbohydrates (Poorter & Villar, 1997). Root carbon concentration was also negatively correlated to aboveground biomass, which indicates that more oxidized carbon compounds also play a role in shoot growth and regrowth after mowing or grazing. Root calcium concentration of the phytometers as well as of the local neighborhood had a positive effect on root dry mass, which points to the importance of roots to store nutrients, as Ca is an important element for plant and especially root growth (Grime et al., 1997; Scheffer, Schachtschabel, & Blume, 2002). Accordingly, root calcium concentration of the phytometers also had a positive effect on leaf and aboveground biomass.

The different predictors for root and leaf growth emphasize the importance to include roots in ecological studies as has been pointed out previously (Bessler et al., 2009; Cadotte, Cavender‐Bares, Tilman, & Oakley, 2009). Up to 90% of the net primary productivity in temperate grasslands can be allocated to belowground organs (Stanton, 1988). Bessler et al. (2009) showed for the Jena‐Experiment that aboveground biomass production increased with increasing species richness, while belowground organs were not affected and concluded that one has to look for responses on both compartments to avoid biased conclusions. Siebenkäs, Schumacher & Roscher (2015) reported a higher allocation to aboveground biomass with increasing fertilization and shade. Thus, roots are not only important components for productivity but also might react differently to neighboring plants or nutrient supply compared to aboveground organs and therefore cannot be neglected when aiming at understanding whole plant growth.

Surprisingly, of the ten environmental variables included in our study, only LUI was a predictor for aboveground biomass in the final model. The higher input of nitrogen in plots with high LUI might enhance the biomass production, as reported by Klaus et al. (2011). Also, Allan et al. (2015) showed a positive relationship between LUI and agricultural production. As the purpose of high LUI is to increase forage production (Foley et al., 2005), the positive relationship was to be expected.

Several traits describing the local neighborhood composition were predictors in the best models of all three performance variables. CWM of root volume had a positive effect on leaf biomass. Communities with a higher root volume might capture more resources, which may be indirectly beneficial also for the phytometers. The negative effect of CWM of LDMC and RMV on root biomass may be an indication that those communities with high LDMC and RMV are more conservative in resource use (Freschet et al., 2010; Pérez‐Harguindeguy et al., 2013), which may reflect environmental conditions but also a community response to disturbance.

The relations of FD are not easy to interpret. On the one hand, communities of the Exploratories with a higher FD coincide with low‐productive communities, such as in the dry grasslands. This could explain the negative effect of FD of root magnesium concentration and FD of shoot dry matter content on root and leaf dry mass, respectively. On the other hand, a higher FD indicates a higher complementary use of resources (Petchey & Gaston, 2002), which should lead to a higher productivity. This was the case for FD of LKC, which positively correlated with leaf dry mass. However, we cannot give a mechanistic explanation for the observed effects. Moreover, as shown by variance partitioning, the overall importance of CWM and FD traits for explaining variation in individual plant performance was low. Furthermore, there still was a high amount of unexplained variation, which is normal when working in natural systems and could be explained by factors we did not account for. Such factors could be, among others, the microbial rhizosphere community, root exudates, or chance events.

## 5 CONCLUSION

We showed that the most important predictors for individual plant performance were the functional traits of the same individual on which biomass was assessed. Among all investigated environmental variables, only land‐use intensity had an influence on plant performance. Additionally, CWM and FD of neighboring plants had a higher explanatory power than the environment. Thus, we were able to show that plant functional traits cannot only be used to predict community productivity but also to predict individual plant performance.

## AUTHOR CONTRIBUTIONS

K.H. and S.D. conducted the field experiment and collected the data. U.J., D.S., and H.B. designed the field experiment. H.B. developed the statistical analysis methods. K.H. and H.B. analyzed the data, K.H. wrote the first draft of the manuscript. All authors read, revised, and approved the manuscript.

## CONFLICTS OF INTEREST

None declared.

## Supporting information

' Click here for additional data file.
