# Supplementary material for: Predicting individual plant performance in grasslands
Source: Ecol Evol. 2017 Sep 22;7(21):8958–65. doi: 10.1002/ece3.3393 (PMC5689490; doi:10.1002/ece3.3393)
Supplement: Supplementary file 1 — ' [file ECE3-7-8958-s001.docx]

**Supporting information** to the paper

Herz, K. et al. Predicting individual plant performance in grasslands. Ecology and Evolution

**Tables**

**Table S1.** Results of the linear mixed effects models after lasso selection. From the predictors shown in Table 1 we first selected the most parsimonious model by lasso procedure using 100-fold cross validation and then included them into a linear mixed effects model, using species and plot as random factors. The results of the final model selection step are presented in Table 1. All variables were scaled by mean and standard deviation prior to analyses. For abbreviations of predictors see Table 1. DM = dry mass

|  | DM roots | | | DM leaves | | | DM above | | |
| --- | --- | --- | --- | --- | --- | --- | --- | --- | --- |
|  | Estimate | *P* |  | Estimate | *P* |  | Estimate | *P* |  |
| (Intercept) | -0.552 | 0.000 | *** | -1.141 | 0.000 | *** | -0.641 | 0.000 | *** |
| LAR | -0.048 | 0.007 | ** |  |  |  | -0.059 | 0.002 | ** |
| RCaC |  |  |  | 0.062 | 0.013 | * | 0.054 | 0.008 | ** |
| RCC | -0.124 | 0.000 | *** |  |  |  | -0.135 | 0.000 | *** |
| RMV |  |  |  |  |  |  | -0.012 | 0.507 | n.s. |
| RSR |  |  |  | -0.296 | 0.000 | *** | -0.465 | 0.000 | *** |
| SLA | -0.037 | 0.029 | * | -0.205 | 0.000 | *** | -0.037 | 0.031 | * |
| LUI |  |  |  | 0.002 | 0.949 | n.s. | 0.031 | 0.129 | n.s. |
| PAP | 0.020 | 0.298 | n.s. |  |  |  |  |  |  |
| pH_1 |  |  |  |  |  |  | -0.002 | 0.918 | n.s. |
| rH_200 |  |  |  |  |  |  | 0.028 | 0.145 | n.s. |
| Ta_10 |  |  |  | -0.028 | 0.325 | n.s. |  |  |  |
| Total_C |  |  |  |  |  |  | -0.011 | 0.623 | n.s. |
| CWM_LDMC | -0.038 | 0.049 | * |  |  |  | -0.047 | 0.024 | * |
| CWM_LNC | 0.001 | 0.959 | n.s. |  |  |  |  |  |  |
| CWM_RCaC | 0.046 | 0.018 | * |  |  |  | 0.046 | 0.049 | * |
| CWM_RMgC |  |  |  |  |  |  | 0.000 | 0.982 | n.s. |
| CWM_RMV | -0.029 | 0.090 | . |  |  |  |  |  |  |
| CWM_RNC |  |  |  | 0.001 | 0.984 | n.s. |  |  |  |
| CWM_RVol |  |  |  | 0.058 | 0.038 | * | 0.031 | 0.123 | n.s. |
| CWM_SLA |  |  |  |  |  |  | 0.016 | 0.469 | n.s. |
| FD_LCaC | -0.021 | 0.250 | n.s. |  |  |  |  |  |  |
| FD_LKC |  |  |  | 0.059 | 0.041 | * | -0.005 | 0.810 | n.s. |
| FD_LNC |  |  |  | 0.003 | 0.902 | n.s. | 0.005 | 0.813 | n.s. |
| FD_RCC |  |  |  | -0.047 | 0.115 | n.s. | -0.015 | 0.430 | n.s. |
| FD_RMgC | -0.030 | 0.096 | . | -0.032 | 0.278 | n.s. |  |  |  |
| FD_SDMC |  |  |  | -0.085 | 0.004 |  |  |  |  |

**Table S2**. Results of the linear mixed effects model. In these models, missing values were excluded only from the predictors in Table 2 which resulted in a higher number of observations. All variables were scaled by mean and standard deviation. Random factors were species and plot. For abbreviations of predictors see Table 1. DM = dry mass, n.a. = not applicable.

|  | DM roots | | DM leaves | | DM above | |
| --- | --- | --- | --- | --- | --- | --- |
| Predictor | Estimate | *P* | Estimate | *P* | Estimate | *P* |
| Intercept | -0.546 | *** | -1.146 | *** | -0.642 | *** |
| LAR | -0.050 | ** |  |  | -0.066 | *** |
| RCaC | 0.062 | *** | 0.065 | ** | 0.064 | *** |
| RCC | -0.125 | *** | -0.300 | *** | -0.129 | *** |
| RSR |  |  |  |  | -0.462 | *** |
| SLA | -0.035 | * | -0.202 | *** |  |  |
| LUI |  |  |  |  | 0.040 | * |
| CWM_LDMC | -0.048 | ** |  |  | -0.050 | ** |
| CWM_RCaC | 0.055 | ** |  |  | 0.066 | *** |
| CWM_RMV | -0.037 | * |  |  |  |  |
| CWM_RVol |  |  | 0.073 | ** |  |  |
| FD_LKC |  |  | 0.068 | ** |  |  |
| FD_RMgC | -0.037 | * |  |  |  |  |
| FD_SDMC |  |  | -0.097 | *** |  |  |
|  |  |  |  |  |  |  |
| n samples | 383 |  | 372 |  | 372 |  |
| R² marg. | 0.287 |  | 0.441 |  | 0.684 |  |
| R² cond. | 0.365 |  | 0.578 |  | 0.720 |  |

**Table S3.** Variance partitioning table of Fig. 1. Values show how much variation (in %) in dry mass of roots, leaves and above-ground organs was explained by which predictor type. DM = dry mass, PT = phytometer traits, Env = Environment, CWM = community-weighted mean, FD = functional diversity. Values below zero were set to zero.

|  | DM roots | DM leaves | DM above |
| --- | --- | --- | --- |
| Traits | 21.12 | 19.30 | 44.73 |
| Env | 0.00 | 1.13 | 0.25 |
| CWM | 0.00 | 0.00 | 0.00 |
| FD | 0.43 | 0.00 | 0.00 |
| Traits+Env | 0.08 | 0.44 | 1.26 |
| Env+CWM | 0.37 | 0.00 | 0.00 |
| Traits+CWM | 0.00 | 0.00 | 0.00 |
| Traits+FD | 0.00 | 0.16 | 2.76 |
| Env+FD | 0.00 | 0.00 | 0.29 |
| CWM+FD | 0.00 | 0.48 | 0.00 |
| Traits+Env+FD | 0.00 | 1.10 | 1.54 |
| Traits+Env+CWM | 1.47 | 0.17 | 0.00 |
| Env+CWM+FD | 2.03 | 1.73 | 0.78 |
| Traits+CWM+FD | 0.63 | 0.17 | 0.12 |
| Traits+Env+CWM+FD | 4.71 | 0.95 | 2.85 |
| Residuals | 72.58 | 80.06 | 48.13 |

**Figures
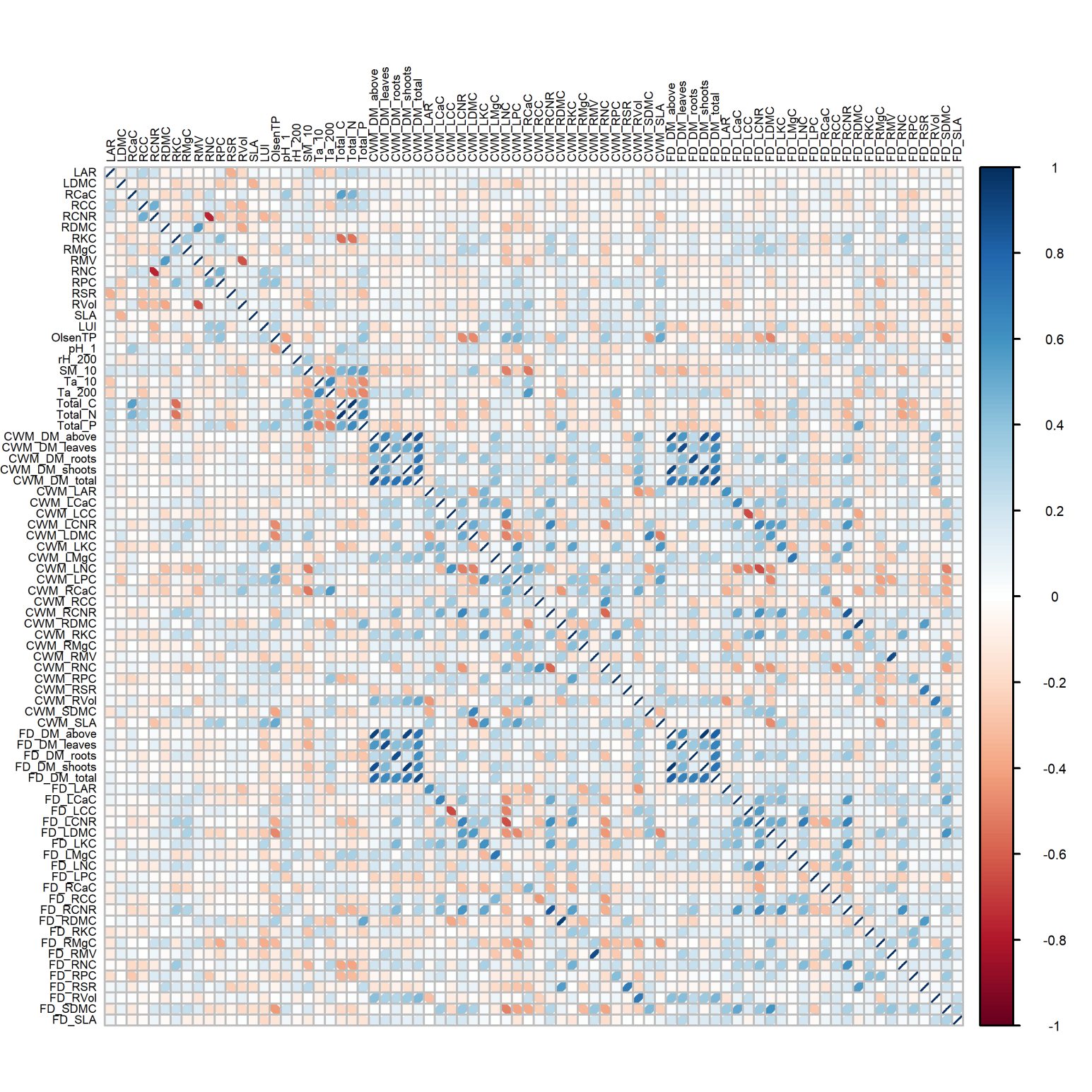
**

**Fig. S1.** Pearson correlation matrix of all variables which were used as predictors for plant performance.

**
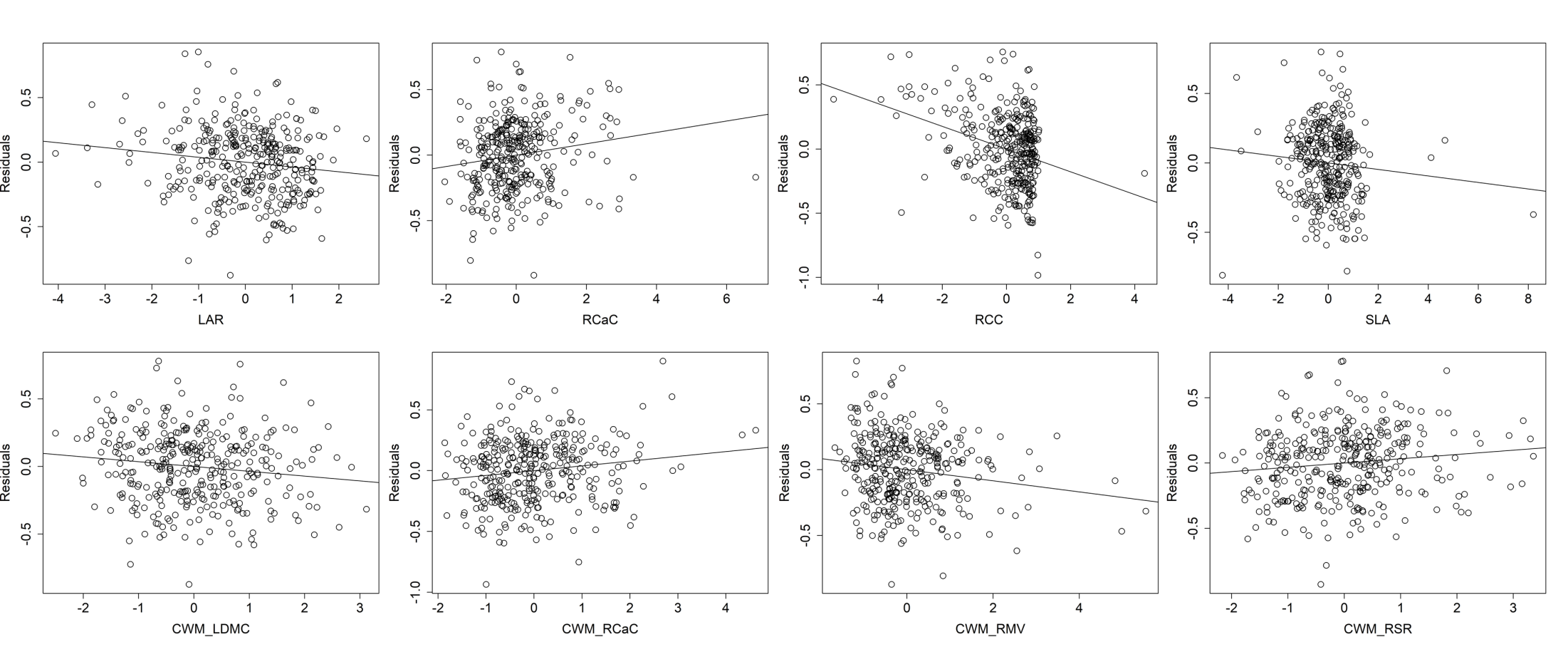
**

**Fig. S2.** Scatterplots of partial effects of the final linear mixed effects model of root dry mass as described in Table 2.

**
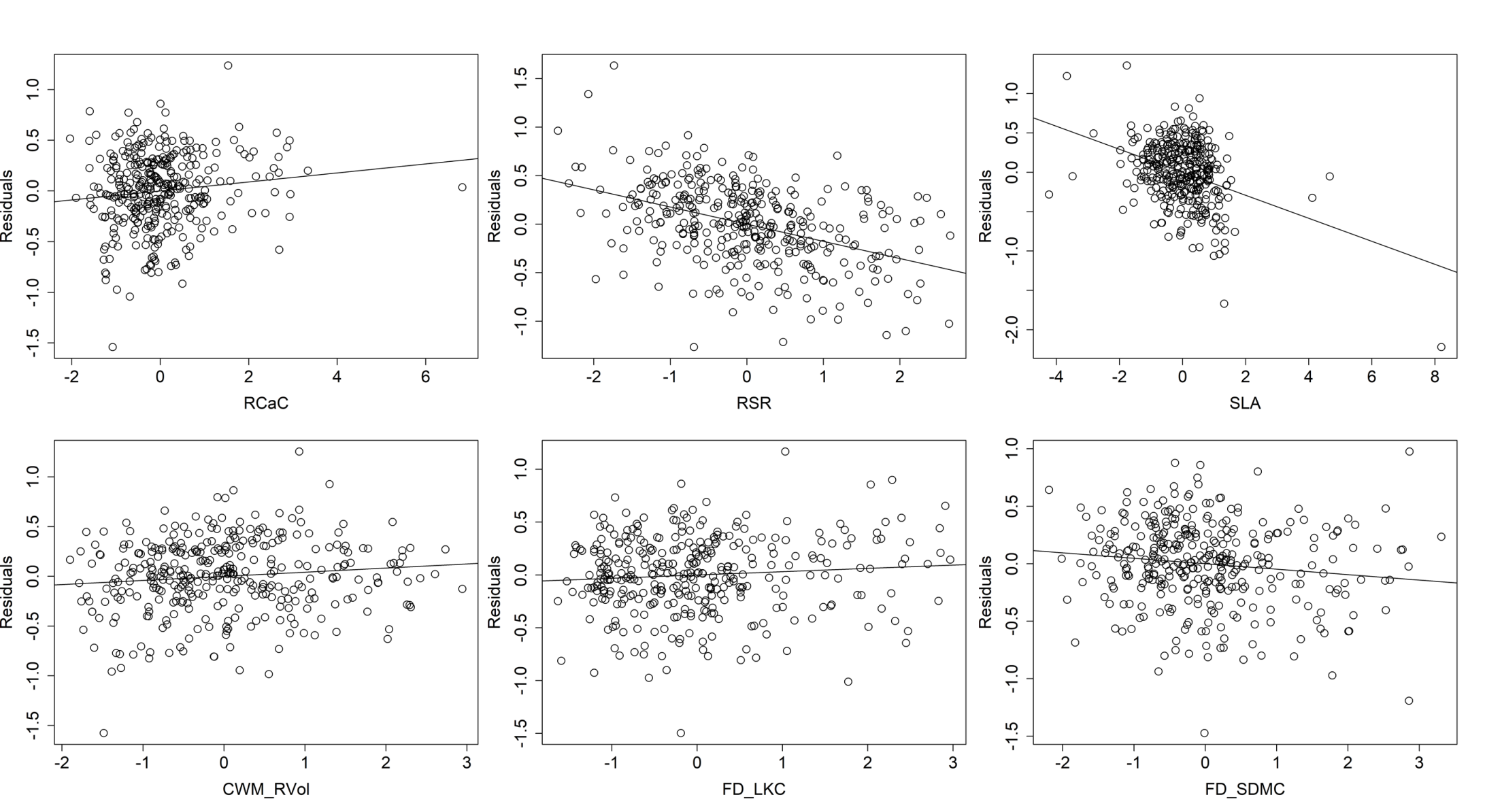
**

**Fig. S3.** Scatterplots of partial effects of the final linear mixed effects model of leaf dry mass as described in Table 2.

**
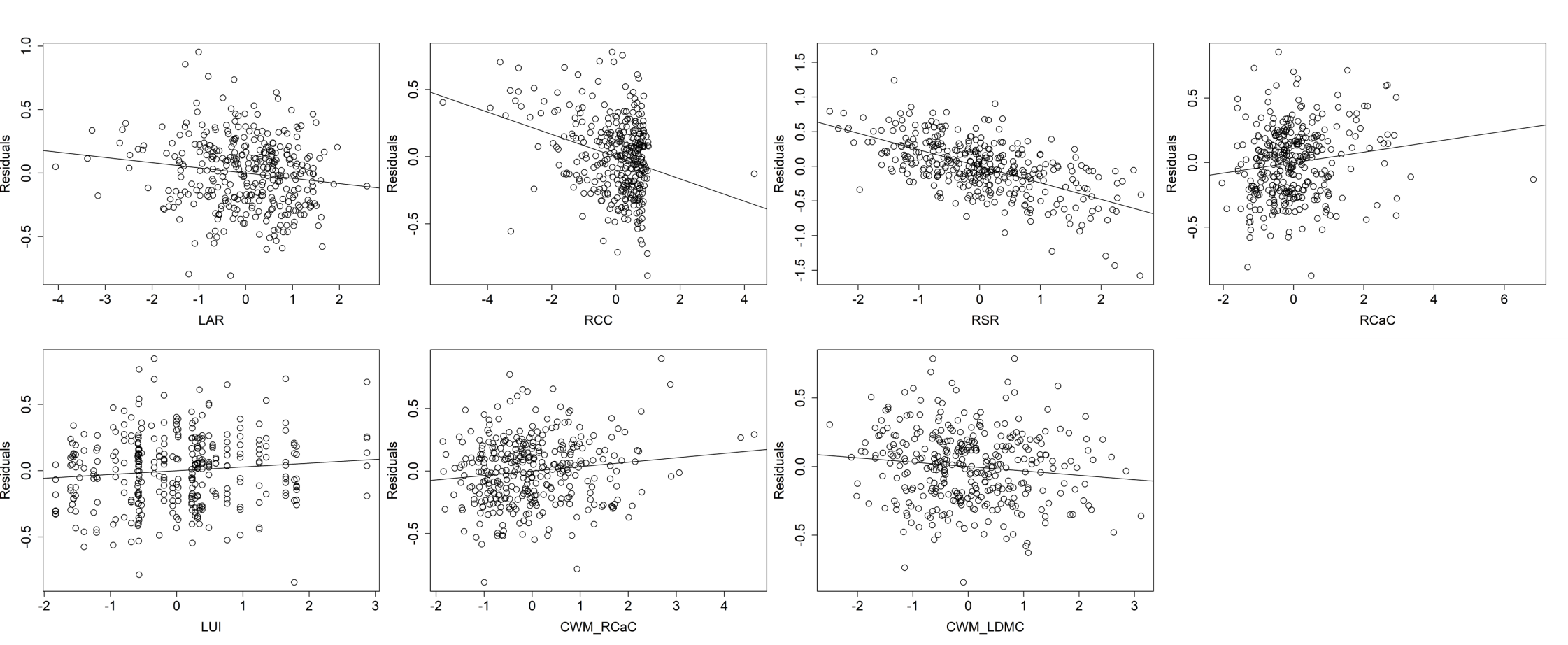
**

**Fig. S4.** Scatterplots of partial effects of the final linear mixed effects model of above-ground dry mass as described in Table 2.
